# Effect of Temperature, Time, and Material Thickness on the Dehydration Process of Tomato

**DOI:** 10.1155/2015/970724

**Published:** 2015-06-15

**Authors:** A. F. K. Correia, A. C. Loro, S. Zanatta, M. H. F. Spoto, T. M. F. S. Vieira

**Affiliations:** ^1^Fruits and Vegetables Laboratory, Department of Agroindustry, Food and Nutrition, University of São Paulo, 13418900 Piracicaba, SP, Brazil; ^2^College of Agriculture “Luiz de Queiroz”, University of São Paulo, 13418900 Piracicaba, SP, Brazil; ^3^Center of Nuclear Energy in Agriculture, University of São Paulo, 13400970 Piracicaba, SP, Brazil

## Abstract

This study aimed to evaluate the effects of temperature, time, and thickness of tomatoes fruits during adiabatic drying process. Dehydration, a simple and inexpensive process compared to other conservation methods, is widely used in the food industry in order to ensure a long shelf life for the product due to the low water activity. This study aimed to obtain the best processing conditions to avoid losses and keep product quality. Factorial design and surface response methodology were applied to fit predictive mathematical models. In the dehydration of tomatoes through the adiabatic process, temperature, time, and sample thickness, which greatly contribute to the physicochemical and sensory characteristics of the final product, were evaluated. The optimum drying conditions were 60°C with the lowest thickness level and shorter time.

## 1. Introduction

The tomato, one of the most scientifically investigated vegetables because of its commercial importance [1], is highly perishable; and postharvesting losses reach 25 to 50%. In tropical countries, there is a loss of 20–50%, from harvesting to consumption [2–5]. Tomato fruit presents high water content, 93–95% [6]. It is low in calories and rich in vitamins A, C, and E and minerals such as calcium, potassium, and phosphorus. In a rank of 10 vitamins and minerals, tomato is the first in terms of contribution in the diet [7, 8].

Brazil is the largest tomato producer in South America, followed by Chile and Argentina. Northeastern region (Pernambuco and Bahia states) accounted for 46% of the production, São Paulo State 30%, and the Cerrado region (Goiás and Minas Gerais states) 24% [9–11].

Drying process consists of the transfer of a fluid in a solid to a nonsaturated gaseous stage [12]. The dehydration in foam layer, lyophilization, drying in a traditional oven and vacuum, and sun drying are among the most widely used methods to process the tomato [13–15]. The removal of moisture must be accomplished in a manner that will be least detrimental to the product quality. Several dehydration processes have been developed to maximize the use of the available conditions for the raw material as well as the energy source used [16]. The dehydration of products stands out as a method to maintain the desired quality for prolonged periods [17, 18]. In addition, drying is a classical method of food preservation; however, due to undesirable changes in quality of dried product pretreatments and drying conditions, studies are necessary [19]. In Brazil, the interest in studies that investigate tomato drying processes is recent, and the dried tomato arrived in the Brazilian market from other countries, namely, Spain and Italy [20, 21].

The consumer's demand for tomato products has increased in recent years [22]. It is increasing rapidly both in domestic and in international markets with major portion of it being used for preparation of convenience food [23]. Tomato and their derivatives are rich in antioxidants and can be considered an important source of carotenoids (lycopene), ascorbic acid, and phenolic compounds [7, 8]. Moreover, the heat increases the bioavailability of lycopene, which is better absorbed by the body when the tomato is cooked, thus, ideal for the consumption of tomato sauces and soups. The industrialization process of the tomato shows that the preparation of sauces, ketchup and others does not destroy lycopene [24–28].

This study evaluated the effects of temperature, time, and thickness of tomatoes slices during the drying process. A central composite design of two and three factorial was applied to investigate the yield.

## 2. Materials and Methods

### 2.1. Dehydration

To investigate the influence of variables on the dehydration of tomatoes, tomato type Carmen cv. (long life) was used. Experiments were conducted using a drying hanger with electric heating, with temperatures ranging between 40 and 80°C, containing 10 perforated trays. The drying hanger has an automatic control of temperature by a digital thermostat coupled with electrical resistance, for automatic temperature stabilization in the cabin of the dryer and forced convection, depending on the air circulation speed of 1.5 m/s. The experiment was conducted in the Food Engineering Laboratory of the Methodist University of Piracicaba (UNIMEP).

**Figure 1:**
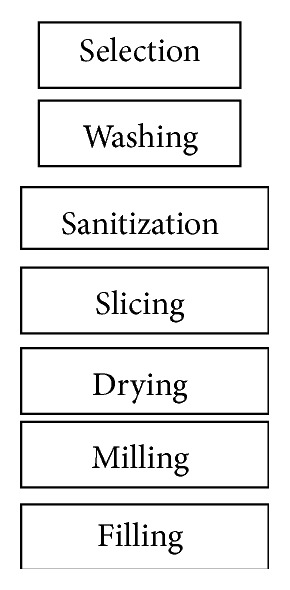
Flowchart of the dehydration process of the tomato.

The variables of the drying process of the tomato were the temperature (°C), time (h), and thickness (mm). The dehydration process of the tomato is shown in Figure 1.

Nearly 1.5 kg of tomatoes was selected which were purchased from the local town market selected according to size, weight, color, strength, and firmness in order to obtain uniformity in the samples. They were washed and soaked for 15 min in an aqueous solution containing 0.2 mL·L^−1^ sanitizer (sodium hypochlorite 2.5%) and were cut into slices with a thickness of 10 mm, 12.9 mm, 20 mm, 27.1 mm, and 30 mm using an industrial slicer, Skymsen PAE-N, which is made of stainless steel and a slicing disc and allows you to set the height, enabling continuous and homogeneous slicing.

Afterwards, the slices were accommodated in trays and placed in a dryer at different temperatures (50°C, 52.9°C, 60°C, 67.0°C, and 70°C) until the final product obtains moisture smaller than 10% because oxidation and browning reactions are the major causes of degradation of dried and intermediate moisture foods [29–31]. Tomatoes have a limited shelf life at ambient conditions and are highly perishable, as previously cited [23]. The estimate of the final product mass was calculated according to the known variables of the initial weight of the product before putting it in the oven and the initial moisture content of the product, according to (1) used by Camargo [21]:

(1) $$\begin{array}{c} M_{f}=M_{i}-\frac{100-U_{i}}{100-U_{f}}, \end{array}$$

where  *M*_*f*_ = final mass of the dried product (g),  *M*_*i*_ = initial mass,  *U*_*i*_ = initial moisture of the product (% wet basis), *U*_*f*_ = final moisture of the product (% wet basis).

The moisture content of the product was determined by a vacuum oven, Marconi MA-30, at a temperature of 70°C until the sample reaches constant weight. The product powder was placed in a container of 1.4-micrometer thickness and thermosealed containing approximately 50 g of the product in each package. The samples were kept at 25°C ± 1 and relative humidity of 60% ± 2.

### 2.2. Factorial Statistical Design

The fresh tomato dehydration processing was performed under different treatments with combinations in thicknesses of tomatoes and dehydration temperature, applying three replicates for each treatment and using the results from averages of repetitions to calculation effect.

During the dehydration process, the loss of product mass by comparing weights of the initial and final volumes in terms of drying time established for 10 h, 15 h and 50 min, 30 h, 44 h and 10 min, and 50 h was measured.

The statistical design of the experiment followed the complete factorial designs 2^2^ and 2^3^ (for two and three variables, resp.). This design provides the best operating conditions of a model by reducing the number of trials when compared to the univariate process of optimization processes.

In this work of tomato dehydration by the adiabatic process of variables, the temperature, time, and thickness of the material were considered, as they make a relevant contribution to the physicochemical and sensorial features of tomato flour. The factor levels were coded as a central point (0), factorial points (−1, +1), and axial points. (−*α*, +*α*). The results of the experiment were analyzed using the Statistica 11 software.

## 3. Results and Discussion

In any drying process, temperature and speed of vaporization depend on the water vapor concentration in the atmosphere [32, 33]. During the conventional air-drying, setting heat and mass transfer results in the removal of moisture by thermal flow with the help of heated air, which flows across the fruit surface. Drying time is shorter with increasing temperatures [34]. Likewise, the temperature influences the process, and the pressure also affects the kinetics of each food type; thus, increasing temperatures reduce the drying time in all cases and this time is decreased further when the drying pressure reduces [35, 36].

Many authors such as Olorunda et al. [37], Hawlader et al. [38], Baloch et al. [39], Shi et al. [40], Zanoni et al. [41], Giovanelli et al. [42], and Telis et al. [43] are dedicated to studying the parameters of drying process. For example, it has been proposed by Zanoni et al. [41] that modification of the operating conditions during air-drying of tomatoes, by using lower temperatures, reducing tomato sample thickness, and promoting partial removal of water (production of intermediate moisture tomatoes), can help to reduce oxidative damage in the final dried product. Another example is the use of osmotic dehydration, which has been suggested by some authors to yield good quality, fully dehydrated, or intermediate moisture products of improved stability [44–47].

Akpinar et al. [48] and Movagharnejad and Nikzad [49] established factors that affect the drying speed and the processing time: food properties and secondary phenomena, linked to the necessity to limit the drying temperature, biophysical and biochemical transformations, and reduction caused by stress during dehydration and nonenzymatic browning reactions. Extreme temperatures and/or times in conventional air-drying can cause serious damage to product flavor, color, and nutrients and reduce the rehydration capacity of the dried product [50, 51]. For example, under high temperatures considerable losses in ascorbic acid content have been reported during the production of dried tomato and tomato pulp [41, 42, 52].

During the conventional air-drying, setting heat and mass transfer results in the removal of moisture by thermal flow with the help of heated air, which flows across the fruit surface. Drying time is shorter with increasing temperatures [34]. Likewise, the temperature influences the process, and the pressure also affects the kinetics of each food type. Thus, increasing temperatures reduce the drying time in all cases and this time is decreased further when the drying pressure reduces [35, 36].

In this experiment, the first variables studied were temperature and drying time, required to carry out the possible combinations of exploratory variables as the experimental design in Tables 1 and 2.

Temperature and time parameters were determined based on the effect of lower temperature in a shorter drying time to reach the final product humidity <10%. Temperatures below 50°C do not promote sufficient displacement of the water vapor from the material to reach the desired humidity and above 70°C volatilization of product components starts. The mass loss of the drying process was obtained by measuring a predetermined volume of product, at regular intervals. The range between the lowest and the highest values for each run was twofold, which demonstrate the importance of applying experimental design at this stage, when methods would take too much time. At the central point, higher intensity of mass loss was observed when compared to results from axial points (Table 2).

**Figure 2:**
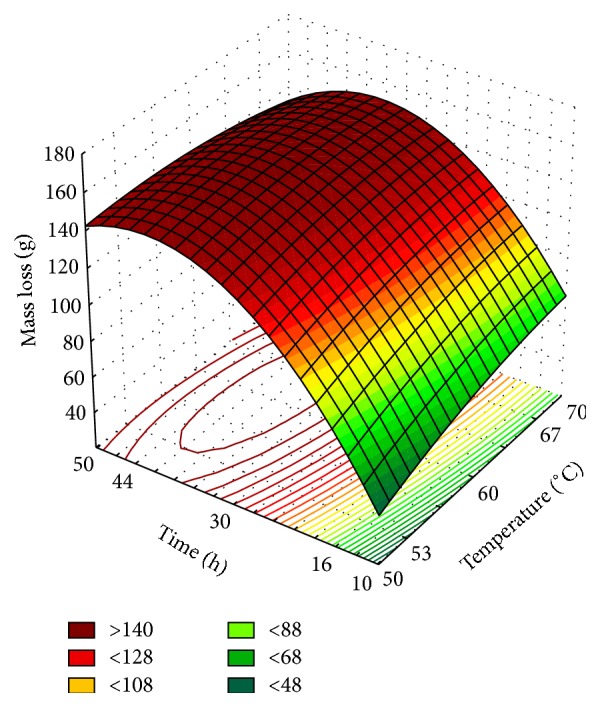
Surface response of tomato dehydration (time × temperature).

The experimental data was used to fit a mathematical model (2). The variance analysis of the regression is shown in Table 3 and adjusted surface response in Figure 2:

(2) Y=146.32+3.5916×T−2.6146×T2+26.601×T′−20.7098×T′2−5.03×T×T′,

where the regression equation coefficients are coefficients, *Y* is the response in question (mass loss), and *T* and *T*′ are coded independent variables (temperature, time, resp.).

Taking into account the *F* calculated higher than threefold *F* tabulated (4.39) at a significance level of 5%, it is possible to state that the model is predictive. Furthermore, either *R* ^2^ or adjusted *R* ^2^ were higher than 0.90. The results shown in Figure 2 allow identifying the relationships between explanatory variables and the quantitative experimental response. The dimensional graph for optimization of tomato dehydration process indicated that the optimal conditions of dehydration process are between 35 h and 44 h at 52–67°C. Therefore, the dehydration process of tomato by stationary system of adiabatic drying, with the factors, internal temperature of the drying chamber and retention time of the product inside the dryer, affects the quantification of the mass loss, represented by the amount of energy enough to evaporate water and remove water vapor from the product surface.

**Figure 3:**
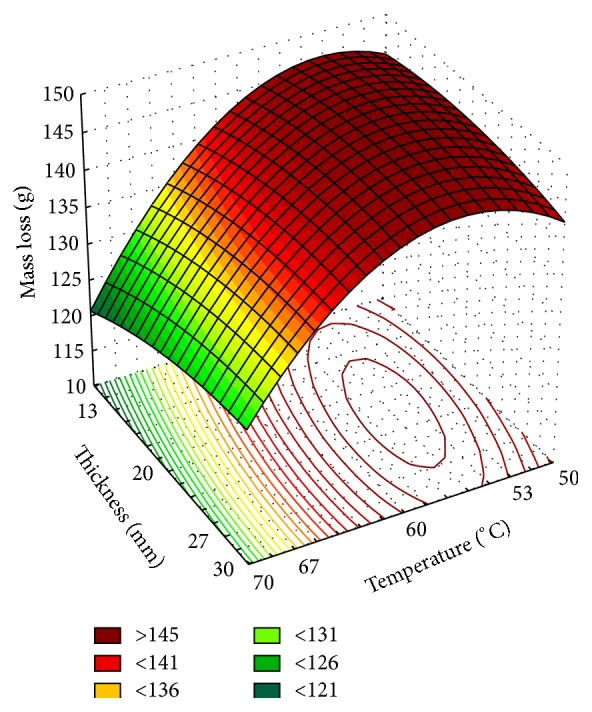
Response surface to tomato dehydration (thickness × temperature).

Another parameter studied was the relationship of temperature and tomato thickness. The results are shown in the experimental design (Tables 4 and 5), ANOVA (Table 6), and adjusted surface response (Figure 3).

The levels of temperature and tomato thickness were established to obtain the variation effect of the geometry of the material, considering the water movement from the inside to the surface of the product by the diffusion mechanism of the liquid and steam due to concentration gradients and temperature. The temperature variation was kept at the same range used in the previous experiment, 50–70°C. For thickness, the axial points were considered as a limitation of the operating process (min. 10 mm and max. 30 mm).

The mass loss (Table 5) of the drying process was obtained by measuring the mass of a predetermined volume of the product until the final moisture content of <10% was attained. The intensity of mass loss was similar among the combinations of the factors and a lower intensity was observed in a combination of variables (1; 1), (−1; 1), and (0; 1.41). Higher intensity mass loss was observed in the variables of the central point.

Adjusted mathematical model (*R* ^2^  0.99) which predicts the effects of temperature and thickness variables and their interactions is

(3) Y=146.34−6.82×T−5.34×T2+1.00×T′′−0.82×T′′2+1.2050×T×T′′,

where the regression equation coefficients are coefficients, *Y* is the response in question (mass loss), and *T* and *T*′′ are coded independent variables (temperature, thickness, resp.).

In the ANOVA, as the *F* calculated for regression was much greater than the tabulated *F* (4.39) at a significance level of 5%, it is concluded that the mathematical model is valid and also can be used for predictive purposes (Table 6).

The surface response showed in Figure 3 indicates the mass loss under the temperature and thickness range applied in drying process of tomatoes. It shows that it is possible to reach the ideal conditions of range of 15–27 mm thickness under 53–58°C, results in accordance with the temperature range for the previous experiment where the variables were temperature × time.

**Figure 4:**
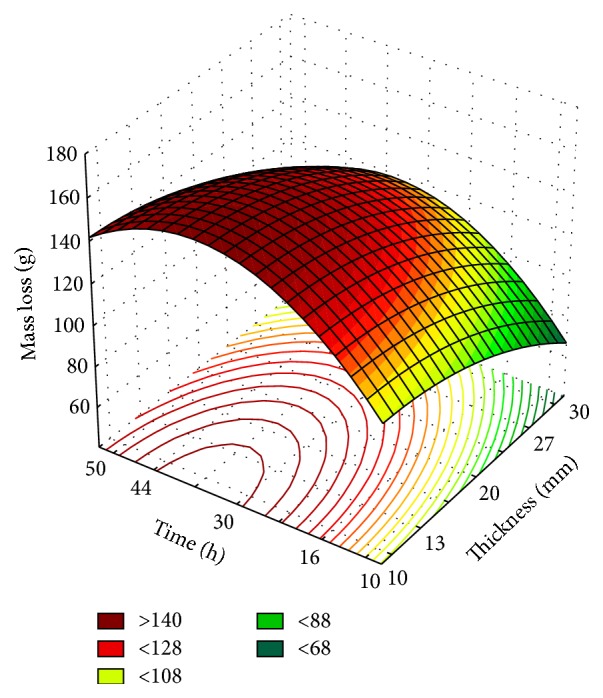
Response surface for tomato dehydration fixing the temperature variation level at 60°C.

In addition to the interactions between two variables, the experiment for the three variables together was performed, thereby obtaining the data for the experimental design (Tables 7 and 8), ANOVA (Table 9), and surface response (Figure 4).

The data in Table 8 indicate that, when related to axial variables of time (10 h and 50 h) and the central points for thickness and temperature, water loss was significantly lower than the other combinations. At extreme low time, there was no effective removal of water vapor from the product surface and at the extreme high time, limits of the thermal flow were exceeded in the moisture removal in the kinetics of tomato dehydration.

The adjusted mathematical model (*R* ^2^  0.99) is presented in the following equation:

(4) Y=145.61−8.62×T′′−3.37×T′′2+9.85×T′−11.42×T′2+2.06×T+1.33×T2−0.05×T′′×T′+2.10×T′′×T−0.165×T′×T,

where the regression equation coefficients are coefficients, *Y* is the response in question (mass loss), and *T*, *T*′, and *T*′′ are coded independent variables (temperature, time, and thickness, resp.).

Table 9 shows that although the model is valid, regression is not predictive, because the *F* value of the regression was not three times greater than the *F*_critical_ value (3.23).

In this model, Box-Behnken design was applied. The three levels of factors, temperature, thickness, and time, fixing the temperature variation level at 0 and applying a factorial 2^2^ with the other two variables, were considered. Figure 4 shows that at fixed temperature of 60°C it is possible to use less thick tomato slices to obtain dried samples in a shorter time, optimizing the process. The temperature of 60°C is viable, since any temperature below this limit cannot effectively remove water, and temperatures above promote the abrupt removal of water causing a possible mischaracterization of the product.

## 4. Conclusion

The tomato powder, as well as most dehydrated foods, should be produced under controlled operating conditions, because the quality of the final product depends on several factors related to the variables of the dehydration process. Thus, the market for dried tomato has increasingly demanded better quality products, which has led to several studies on the matter.

The models of adiabatic drying of tomatoes were predictive, as they showed *R* ^2^ above 0.7 and when subjected to the analysis of variance, except for the last adjusted model, which requires further studies for its validation. Optimal conditions in the dehydration process were 35–44 h at 52–67°C with tomato slices between 15 and 27 mm thickness. When the temperature is adjusted at 60°C, a thinner slice of tomato can be used, reducing the processing time.

## Figures and Tables

**Table 1:** Coded values and corresponding actual values used in the first experimental design.

Exploratory variable	Level of variation				
	−1.41 (−*α*)	−1	0	1	1.41 (*α*)
Temperature (°C)	50	52.9	60	67.0	70
Time (h)	10	15.82	30	44.18	50

**Table 2:** Central composite design (CCD) with observed response for mass loss (g).

Run	Exploratory variables		Mass loss (g)
	Temperature (°C)	Time (h)	
1	−1	−1	83.36
2	1	−1	104.67
3	−1	1	145.54
4	1	1	146.73
5	−1.41	0	141.88
6	1.41	0	146.24
7	0	−1.41	69.81
8	0	1.41	146.36
9	0	0	146.31
10	0	0	146.28
11	0	0	146.40
12	0	0	146.22

**Table 3:** Analysis of variance (ANOVA) for tomato mass loss subject to different times and temperatures during drying process.

FV	SQ	GL	MQ	*F*
Regression	8584.553	5	1716.911	98.87475
Residue	104.187	6	17.3645	
Lack of adjustment	104.17	3	34.72333	6127.647
Pure error	0.017	3	0.005667	

Total	8688.74	11		

**Table 4:** Coded values and corresponding actual values used in the second experimental design.

Exploratory variable	Level of variation				
	−1.41	−1	0	1	1.41
Temperatures (°C)	50	52.9	60	67.0	70
Thickness (mm)	10	12.9	20	27.1	30

**Table 5:** Central composite design (CCD) with observed response for tomato mass loss (g).

Test	Exploratory variables		Mass loss (g)
	Temperature (°C)	Thickness (mm)	
1	−1	−1	145.59
2	1	−1	146.38
3	−1	1	129.42
4	1	1	135.03
5	−1.41	0	145.22
6	1.41	0	146.35
7	0	−1.41	146.35
8	0	1.41	127.25
9	0	0	146.34
10	0	0	146.32
11	0	0	146.31
12	0	0	146.41

**Table 6:** Analysis of variance (ANOVA) for tomato mass loss subjected to different thickness and temperatures during drying process.

FV	SQ	GL	MQ	*F*
Regression	567.242	5	113.4484	56.0551
Residue	12.14324	6	2.023873	
Lack of adjustment	12.1428	3	4.047587	25563.71
Pure error	0.0005	3	0.000158	

Total	579.3853	11		

**Table 7:** Coded values and corresponding actual values used in the third experimental design.

Exploratory variable	Level of variation				
	−1.68	−1	0	1	1.68
Thickness	10	12.9	20	27.1	30
Time	10	15.82	30	44.18	50
Temperature	50	52.9	60	67.0	70

**Table 8:** Box-Behnken design with observed response for tomato mass loss (g).

Test	Exploratory variables			Mass loss (g)
	Thickness	Time	Temperature	
1	−1	−1	−1	143.85
2	1	−1	−1	130.92
3	−1	1	−1	145.59
4	1	1	−1	122.8
5	−1	−1	1	144.97
6	1	−1	1	130.8
7	−1	1	1	136.39
8	1	1	1	131.68
9	−1.68	0	0	143.62
10	1.68	0	0	127.99
11	0	−1.68	0	69.81
12	0	1.68	0	81.36
13	0	0	−1.68	141.88
14	0	0	1.68	146.31
15	0	0	0	146.31
16	0	0	0	146.29
17	0	0	0	146.25
18	0	0	0	146.2

**Table 9:** Analysis of variance (ANOVA) for tomato mass loss during drying process.

FV	SQ	GL	MQ	*F*
Regression	6975.588	8	871.9485	5.94
Residue	1320.81	9	146.7567	
Lack of adjustment	1320.803	6	220.1339	93343.0
Pure error	0.007	3	0.002358	

Total	8296.398	17		

## Practical Application

The practical application is to study the drying process to optimize the dehydration process for tomatoes fruits.
